# Laparoscopic splenectomy for tuberculous abscess of the spleen

**DOI:** 10.4103/0972-9941.68582

**Published:** 2010

**Authors:** Deepraj Bhandarkar, Avinash Katara, Manu Shankar, Gaurav Mittal, Tehemton E Udwadia

**Affiliations:** Division of Minimal Access Surgery, P D Hinduja National Hospital, Veer Savarkar Road, Mahim, Mumbai 400016, India; 1 Department of Surgery, Fortis Escorts Hospital and Research Centre, Neelam Bata Road, Faridabad, India

**Keywords:** Abscess, laparoscopy, spleen, splenectomy, tuberculosis

## Abstract

Abscess of the spleen is an uncommon clinical entity and a tuberculous abscess is particularly rare. Although image-guided aspiration has been reported, splenectomy is the preferred modality of treatment. We report a 32-year-old female diagnosed to have a large, multilocular splenic abscess during investigation of a pyrexial illness. Her haemoglobin was 9.8 gm%, ESR 100 mm/1^st^ hour and she was HIV negative. She had been on anti-tubercular chemotherapy (started elsewhere) for 2 months but had shown poor response. A laparoscopic splenectomy undertaken using four-ports was challenging due to the presence of perisplenitis and adhesions in the splenic hilum. Also, fundus of stomach densely adherent to the upper pole of the spleen required stapled resection. Postoperatively, she developed a low-output pancreatic fistula that resolved with conservative treatment within a week. Histopathology of the spleen confirmed tuberculosis. She responded well to anti-tubercular chemotherapy and remains well 3 years later.

## INTRODUCTION

The role of laparoscopic splenectomy (LS) is well established in the treatment of haematological conditions but infective disorders form an uncommon indication. We report a patient with tuberculous abscess of the spleen who underwent LS.

## CASE REPORT

**Figure 1:**
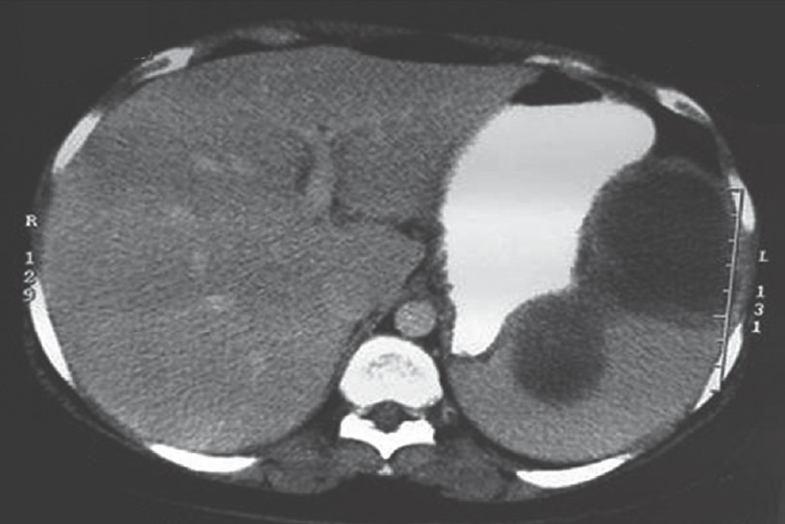
CT scan showing a large, multilocular splenic abscess

A 32-year-old woman who had low-grade pyrexia for 3 months was investigated elsewhere and referred to us with a diagnosis of a splenic abscess. Clinically, she had a mildly tender splenomegaly extending 4-cm below the costal margin. The investigations showed haemoglobin to be 9.8 gm% and ESR to be 100 mm/1^st^ hour. All the biochemical parameters and chest x-ray were normal and she was HIV-negative. An echocardiogram was normal. Her computerized tomography (CT) scan showed a large, multilocular splenic abscess [Figure 1]. She had received two months of anti-tubercular chemotherapy (started elsewhere) but had responded poorly to it. She was administered pneumococcal and meningococcal vaccines in preparation of LS.

**Figure 2:**
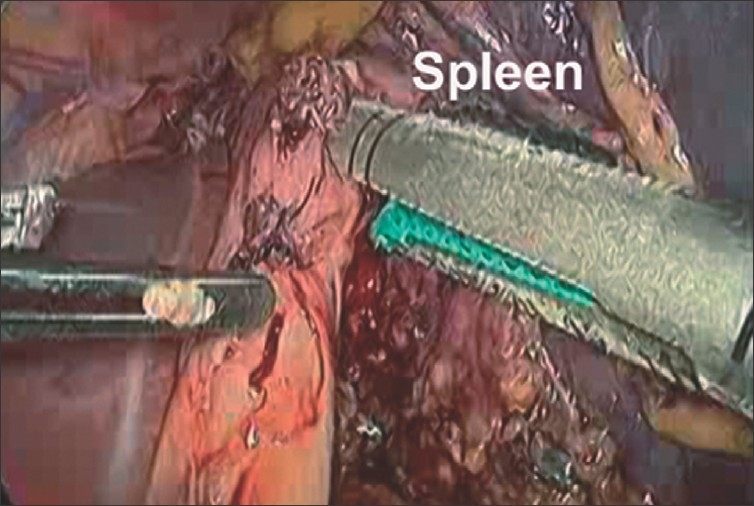
Stapled resection of stomach adherent to the spleen

Surgery was performed under general endotracheal anaesthesia with the patient in left lateral decubitus position. A nasogastric tube and an indwelling urinary catheter were in situ. The first 10-mm port was placed midway between the left costal margin and the umbilicus by the open method. Another 10-mm and two 5-mm ports were positioned around the first port. Omentum adherent to the spleen was divided using an ultrasonic shears (Ethicon Endosurgery, Cincinnati, OH, USA). The lesser sac was entered and the splenic artery was dissected and divided after clipping. The short gastric vessels were divided using the ultrasonic shears. Two firings of a 45-mm endoscopic linear cutter device (Endo GIA, Ethicon Endosurgery) were used to resect the gastric fundus densely adherent to the spleen [Figure 2]. Dense adhesions at the splenic hilum precluded identification and dissection of the splenic vein. A 10-mm Ligasure vessel sealing device (Covidien, Boulder, Colorado, USA) was used for control of the hilum and for division of the posterior as well as diaphragmatic attachments of the spleen. The excised spleen was placed in an impervious retrieval bag and extracted after enlarging the site of the telescopic port. A tube drain was placed and the fascia at the extraction site was closed with non-absorbable suture. The skin was approximated with subcuticular suture. The total operative time was 230 min and blood loss 700 ml. Postoperatively, the drain yielded around 100 ml of amylase-rich fluid for the first 3 days. The patient was kept nil per oral and administered parenteral nutrition and subcutaneous octreotide. The drain was removed on the seventh day once the output reduced to 20 ml/24h and ultrasonography showed no residual collection. Histopathology of the spleen confirmed tuberculosis. She responded well to anti-tubercular chemotherapy and remained well 3 years later.

## DISCUSSION

Splenic abscess is an uncommon clinical condition, and autopsy studies have established its incidence at between 0.14% and 0.7%.[1] The cause of splenic abscess could be metastatic infection, superinfection following ischemia or infarction due to red blood cell abnormalities, trauma, contiguous infection or immunodeficiency. The organisms commonly responsible include *Streptococcus, Staphylococcus, Enterobacteriaceae, Chlamydia pneumoniae, Brucella, Clostridium*, or fungi. Tuberculosis is an even rare cause of splenic abscess but incidence of splenic tuberculosis is on the rise in immunodeficient patients.

Tuberculous abscess of the spleen is difficult to diagnose due to the paucity of specific findings. Splenomegaly, fever, anorexia, weight loss, abdominal pain, ascites, cough and lymphadenopathy may be present in varying combinations. Spontaneous rupture of the tuberculous splenic abscess (both in HIV-positive patients and in normal individuals) may present with acute abdomen.[2] Imaging studies play an important role in establishing the diagnosis of splenic tuberculosis. Common ultrasonographic findings include single or multiple focal hypoechoic lesions, representing granulomas. Hyperechoic lesions, isolated splenomegaly, perisplenic abscess and hepatic lesions can also be detected.[3] CT scan is superior to ultrasonography in the assessment of these patients as it is able to localize sub-centimetre lesions and give better anatomic information about the perisplenic area and contiguous viscera. Early and judicious use of imaging in appropriate clinical settings helps the clinician identify and treat a splenic abscess early and avoid the mortality that may ensue from a late diagnosis. Rarely, laparoscopy may be needed to establish the diagnosis of splenic tuberculosis as was reported by Meshikhes *et al*. in a patient with pyrexia of unknown origin.[4] Although a percutaneous biopsy is perhaps preferable to a laparoscopic biopsy, the latter carries the advantage of obtaining secure haemostasis under direct vision.

Several authors have reported the use of aspiration of splenic abscesses under ultrasonographic or CT guidance in a select group of patients with the aim of avoiding splenectomy and preserving immunological function.[5–7] Although an attractive option, image-guided drainage is only likely to succeed when (a) the collection is unilocular or bilocular with a discrete wall and no internal septations, (b) when its content is liquefied enough to be drained and (c) it is located at the periphery or at the middle or lower pole of the spleen.[6]

Traditionally open splenectomy has been found to be the most effective and definitive procedure for most patients with splenic abscess. The mortality rates of this surgery are reported to vary from 0% to 16.9% and the morbidity rates from 28% to 43%.[7,8] The high rates of morbidity and mortality are likely to be a reflection of the predisposing disease states.

LS for splenic abscess is sparsely reported in the literature, and there do not appear to be any cases describing this procedure for tuberculous abscess of the spleen. Carbonell *et al*. reported a series of four patients undergoing LS for splenic abscess.[9] There were no postoperative complications or deaths. Simsir *et al*. reported two patients with splenic abscess due to infective endocarditis who underwent LS followed by valve replacement during the same hospitalization.[10] In suitably located splenic abscess, splenic conservation may be attempted. De Greef *et al* performed a laparoscopic partial splenectomy in a 12-year old girl with *Salmonella*-induced splenic abscess in the upper pole.[11] The short gastric vessels and upper segmental branches were coagulated to obtain a clear line of demarcation; radiofrequency (RF) ablation was used at the demarcation line and the parenchymal division was done using ultrasonic shears. Such a procedure may be feasible only rarely due to the dense adhesions that are invariably present. It is not uncommon to have a part of the stomach densely adherent to the spleen as in our patient who required a partial resection of the gastric fundus. Dissection of the splenic vessels within the hilum covered with adhesions and engulfed in inflammation could be challenging, and control of the vessels using either an energy source such as the bipolar vessel sealing device or a vascular stapler may be required. In our patient, the tail of the pancreas was likely injured during control of the hilum resulting in the postoperative pancreatic fistula. It is prudent to exercise caution in manipulating a spleen with abscess so as to prevent spillage of its contents and always retrieve it after placing it in an impermeable bag.

In conclusion, LS is a feasible alternative to open splenectomy in patients with tuberculous abscess of the spleen, but this technically demanding procedure is best undertaken by experienced laparoscopic surgeons. In this clinical setting, LS is likely to result in a higher postoperative morbidity than when performed for other indications.
